# Developing and prioritising strategies to improve the implementation of maternal healthcare guidelines in South Africa: The nominal group technique

**DOI:** 10.4102/phcfm.v14i1.3184

**Published:** 2022-08-04

**Authors:** Thifhelimbilu I. Ramavhoya, Maria S. Maputle, Tinyiko R. Lebese

**Affiliations:** 1 Department of Advanced Nursing Science, Faculty of Health Sciences, University of Venda, Thohoyandou, South Africa; 2 Department of Nursing Sciences, Faculty of Health Sciences, University of Pretoria, Pretoria, South Africa

**Keywords:** strategies, improve, provision, maternal guidelines, BOEM

## Abstract

**Background:**

In South Africa, maternal healthcare guidelines are distributed to primary health care (PHC) facility for midwives to refer and implement during maternal healthcare services. Different training was offered for the use of maternal care guidelines. However, poor adherence and poor implementation of guidelines were discovered.

**Aim:**

This study aimed to develop and prioritise strategies to improve the implementation of maternal healthcare guidelines at PHC facilities of Limpopo province, South Africa.

**Method:**

Strengths, weaknesses, opportunities and threats analysis and its matrix together with the nominal group technique were used to develop the current strategy. Midwives, maternal, assistant and operational managers from PHC facilities of the two selected district of the Limpopo province were selected. Criterion-based purposive sampling was used to select participants. Data collection and analysis involved the four steps used in the nominal group technique.

**Results:**

Strategies related to strengths and weaknesses such as human resources, maternal health services and knowledge deficit were identified. Opportunities and threats such as availability of guidelines, community involvement and quality assurance as factors that influenced the provision of maternal healthcare services were identified.

**Conclusion:**

Researchers formulated actions that could build on identified strengths, overcome weaknesses such as human resources, explore opportunities and mitigate the threats such as quality assurance. Implementation of the developed strategies might lead to the reduction of the maternal mortality rate.

## Introduction

South Africa, like other countries, is utilising maternal healthcare guidelines derived from World Health Organization (WHO) to provide quality maternal healthcare to pregnant women.^1^ With the management of pregnant women, the South African maternity healthcare guidelines incorporated the preventable conditions, the management of pregnant women during antenatal care (ANC), labour and postnatal management.^2^ The maternity guidelines are one manual for clinics, community health centres and district hospitals for better and easy use by midwives.^2^ The Maternal Health Directorate in the Department of Health distributes the manual to every healthcare facility in the Limpopo province. The midwives utilise these guidelines to refer and implement during maternal healthcare services. The directorate is offering training on updates regarding the implementation of guidelines; however, not all midwives are reached.

In qualitative studies conducted by Ramavhoya^3,4^ on 18 midwives, their experiences in the management of pregnant women with hypertensive disorders and post-partum haemorrhage (PPH) were explored. The two studies revealed that managing pregnant women with pre-eclampsia, eclampsia and post-partum haemorrhage was a difficult task that resulted in frustrations and confusion. It required the management of the women with the help of another midwife, hence the shortage of midwives as alluded in the study had a negative effect in the management of women with either eclampsia or PPH.^3,4^ The steps in the management of these conditions were indicated on the maternity healthcare guidelines and illustrated on protocols pasted on the walls of the delivery room of each primary health care (PHC) facility. Midwives experienced difficulty in following the guidelines as some of the emergency obstetric conditions such as PPH occurred abruptly without the midwives expectations and needed urgent attention to save the women’s lives. This was also influenced by the knowledge and skills that midwives possess. In the results of a quantitative study conducted on 200 midwives, it was revealed that 34.5% of the midwives who participated in the study were not trained on the essential management of obstetric emergencies (ESMOE).^5^ Another factor that negatively impacted the management of women discovered from the survey was non-compliant in ambulance services which led to late initiation of the management by the second level of care, leading to an increased maternal mortality rate (MMR).

The quantitative descriptive cross-sectional study was conducted on 60 managers to determine the support offered to midwives in the implementation of maternal healthcare guidelines. The study revealed that staff shortage, material resources, inadequate supervision and monitoring of midwives by facility managers and district maternal health care managers (DMHCMs) and unsatisfactory facility visits by DMHCMs contributed to the substandard implementation of the guidelines. Although support through training was highly noted, it did not cover all midwives.^6^

Talking about the studies conducted by Fulmer, Braddick indicated that unfair distribution of resources, infrastructure and capabilities, skills and competency levels of some midwives were amongst the aggravating factors influencing high maternal morbidity and mortality rate.^7,8^ The study conducted on experiences of midwives/accoucheurs in the implementation of pregnancy human immunodeficiency virus (HIV) guidelines on 18 midwives revealed that midwives experience work overload caused by many procedures that were performed in pregnant women who tested HIV positive during their initial visits and many registers that midwives had to complete. This leads to long waiting times for other patients. This study further indicated that midwives were adhering to the Prevention of Mother-to-Child Transmission (PMTCT) guideline when providing care to pregnant women affected with HIV.^9^ The implementation of (PMTCT) guidelines for HIV resulted in a decrease of mother-to-child transmission rate and MMR because of HIV infections as compared to the other preventable conditions.^10^ Although most midwives followed the HIV guidelines during the provision and care of pregnant women, adherence to maternal care guidelines in women with hypertensive disorders and PPH was poor. These results were similar to studies conducted by Roberts, WHO and the Saving Mothers’ Report of 2014–2016. Literature from these studies indicated that non-adherence and poor implementation of maternal healthcare guidelines resulted in high morbidity and mortality rate.^11,12,13^ Similar to weeks results, it was found that only few participants executed maternal care guidelines correctly; hence the majority of midwives were implementing the guidelines partially.^6,14^ If all midwives were implementing the guidelines correctly, the health facilities, healthcare professionals and the community represented by patients would benefit.^7,15^ Maternal morbidity and mortality rate would decline to 5.5% annually as indicated in the Sustainable Development Goals by 2030.^8^ It was against this background that researchers developed and prioritised strategies to improve the implementation of maternal healthcare guidelines at PHC facilities of Limpopo province, South Africa.

## Methods

### Research designs

In developing the current strategies, researchers used the steps of Pearce,^16^ which are, strengths, weaknesses, opportunities and threats (SWOT) analysis.^16^ The study took place in three phases. Phase one was the empirical phase which was quantitative and qualitative; phase two was strategy development and phase three was validation of developed strategies. Five articles were published from the findings of phase 1 on post-partum haemorrhage, Hypertensive disorders in pregnancy, HIV and support offered by managers to midwives in implementing maternal healthcare guidelines^3,4,5,6,9^ which formed the basis of the current background of this article. From the results obtained in phase 1, researchers conducted SWOT analysis to identify key issues and organised them in a SWOT matrix.

The Nominal Group Technique (NGT) was utilised for the group to identify and prioritise strategies to address the issues from the SWOT analysis and to elaborate on the actions needed to implement these strategies (phase 2). During this process, the researchers collected both qualitative and quantitative data where ideas were brainstormed, listed and discussed followed by ranking through voting and agreed upon by all members of the group.

The population suitable for phase two were Provincial and District maternal healthcare managers, operational managers (OMNs), assistant managers and midwives working at the PHC facilities. The sampled members of NGT were given the SWOT matrix a week before the strategy development meeting to familiarise themselves with the matrix and for the group to have a voice in the development and prioritisation of the current strategies. This also ensured that participants had scientific evidence of phase one and their experiences in rendering maternal healthcare services played an essential role in developing the current strategies. Nominal Group Technique was appropriate for this study as it allows participants to participate without discrimination and intimidation from other participants, and the consensus is reached amicably.^17,18^ The results of participation by the NGT led to the development of the current strategies. This article will report the results of phase two, which is the development and prioritisation of strategies to improve midwives’ implementation of maternal healthcare guidelines at the PHC facilities of the Limpopo province. Phase three of the study was validation of the developed strategies conducted in the same province with midwives and OMN who did not participate in the NGT process. The process of validation will be reported in another article.

### Study setting

This study was conducted in the PHC facilities of the Vhembe and Mopani districts in the Limpopo province, where maternal health guidelines should be implemented. The two districts form borders with neighbouring countries of Zimbabwe, Botswana and Mozambique and are experiencing a high influx of clients from Zimbabwe and Mozambique. Both districts are rural and consist of 223 PHC facilities including community health centres. Most of the PHC facilities consist of 4–7 professional nurses with 4 or 5 midwives and fewer advanced midwives. Fourty (40) to 50 pregnant women per month are seen in most clinics and 50–60 for community health centres. Deliveries at both levels of care range from 20 to 40 per month. Some facilities render 24-h services (call system) with community health centres operating day and night shifts, whilst others offer 12-h services. The PHC facilities use the supermarket approach to provide comprehensive PHC packages. Although, currently, the PHC facilities are using an ideal clinic realisation approach which involves three streams grouped as mother and child, minor ailment and chronic care streams.

### Population and sampling

The population consisted of OMNs, DMHCMs, provincial maternal health care managers, assistant managers (AM) and midwives of the two selected districts of the Limpopo province. The criterion-based purposive sampling method was used to select maternal healthcare services’ experts with more than five years of working experience in maternal healthcare and participated in phase one of the study.

One provincial maternal healthcare manager (MHCM) was selected as she oversee the programme within the province. Four midwives (two from each district) were selected as they are providers of maternal healthcare guidelines, two OMN (one from each district) as supervisors of midwives in PHC facilities. The two DMHCMs oversee maternal healthcare services within each district and two AMs (one from each district) as overall supervisors of PHC facilities. They also monitor maternal healthcare services in the local area. As the NGT usually involves the selection of 5–12 participants,^18^ with the development and prioritisation of the current strategies, a total of 11 participants were sampled to form part of the NGT.

### Data collection

The researchers sent invitations to all who participated in the NGT. Eleven experts in maternal healthcare services met with the moderator and her assistant a week later on the date, time and place agreed with the group. Together with the researcher and her assistants, experts in maternal healthcare services worked as a team of researchers in developing the current strategies. The group was given the SWOT analysis matrix to familiarise themselves with the matrix’s content and help them make a final decision. On the meeting day, the researcher acted as the moderator and facilitated the group. The moderator welcomed the group and explained the purpose of the meeting, which was to develop and prioritise strategies that will improve midwives’ implementation of maternal healthcare guidelines by midwives based on the results of phase 1 inclusive of the five articles. The moderator presented the SWOT analysis matrix to the group clarifying any misunderstandings. The moderator then divided the group into four and instructed each group to discuss and write down their ideas following building on strength, overcoming weakness, exploring opportunities and minimising threats (BOEM) that can be used in the development of the current strategies. Participants were given 20 min to brainstorm the strategies using BOEM actions in silence. The moderator asked each group one by one the strategies that they wrote whilst the moderator’s assistant was jotting them down on the flip chart, for which no discussion occurred. This process was repeated until all the ideas were written down. Written ideas were discussed one by one so that group members get clarity of the listed ideas and add their inputs that will uplift the listed strategies.

### Data analysis

Data analysis was performed simultaneously with data collection as the NGT allowed the process to be performed simultaneously.^17,18,19^ The ranking sheet with listed ideas from the flip chart was prepared by the moderator’s assistant and given to each participant to think carefully, select and prioritise the most important listed items independently. Participants were asked to individually rank the seven most important strategies and to assign the score of 7 (the most important) to 1 (the least important). Twenty minutes’ time was allocated for this activity. Ranking sheets were collected from participants and the moderator asked one member to read them one by one, with the moderator writing results of each participant on the ranking sheet prepared on the flip chart for all participants to see. Total scores were calculated and indicated towards the end of each listed idea. The top seven strategies were then identified from the total scores and categorised according to the aspects of the SWOT analysis they addressed. Development and prioritisation of strategies’ session with the group took 4 h. The ranked strategies and their actions are noted in the results section below in Tables 1 and 2.

**TABLE 1:** Priority strategies to building on strength and overcoming weaknesses.

Ranking	Strategies on strength and weakness	Total scores	Actions to be taken to build on strength and overcome weaknesses
1	Availability of human resources	74	MHCM to develop scheduled site visits to support PHC facilities Integration of programmes for OMNs to supervise and monitor the implementation of maternal healthcare services Filling of vacant posts and remunerations for seconded OMNs Hiring of more midwives who will support each other in managing pregnant women Clubbing of PHC facilities together to relieve the shortage of midwives
2	Knowledge deficit leading to difficulty and frustrations amongst midwives	70	Allocation of advanced midwives to each PHC facility Capacity building through various training programmes, including ESMOE Allocation of two midwives to manage pregnant women
3	Provision of maternal healthcare services	69	Referral of pregnant women for further management HIV counselling offered, need for human resource and test kit. Consolidation of registers to reduce the workload Purchasing of ambulances designated for maternal health Provision of anti-eclamptic drugs in facilities with shortages Inclusion of drugs that prevent eclampsia, magnesium sulphate and uterotonics on the reporting system To offer 24-h services to promote access to maternal healthcare services Designing maternal obstetric units and building maternity hospitals for high-risk women and those who are lost to follow-up and poor access. Provision of park homes in facilities with fewer consulting rooms

MHCM, maternal health care manager; OMN, operational manager; ESMOE, essential management of obstetric emergencies; HIV, human immunodeficiency virus; PHC, primary health care.

**TABLE 2:** Priority strategies to explore opportunities and minimising threats.

Ranking	Strategies	Total scores	Actions to be taken
4	Quality assurance	68	Monthly facility assessment and peer reviews to be conducted Monthly perinatal meetings conducted Quarterly value clarification/workers for a change meeting Client satisfaction surveys to be conducted Clinical audits of records to be conducted Change attitude midwives and women to promote co-operation Incorporation of cell phone remainders in the Mom-Connect programme
5	Availability of PMTCT, maternity care guidelines and protocols	66	Positive attitudes of midwives towards guidelines Packaging all PHC guidelines electronically
6	Community involvement	65	Training of home-based carers and life orientation on maternal health issues Accompaniment of pregnant women to PHC facilities for delivery
7	Reduction of waiting times	63	Consolidation of registers to avoid duplications; Use of appointment strategies Provision of park homes to communities without PHC facilities

PMTCT, prevention of mother-to-child transmission; PHC, primary health care.

## Results

### Priority strategies and their actions of building on strengths, overcoming weaknesses, exploring opportunities and minimising threats

Tables 1 and 2 depict the results of the developed and prioritised strategies with the NGT group as researchers following SWOT analysis where seven strategies under SWOT were identified. The strategies were ranked in order of priority as indicated by the group. The strategies were developed and actions that were building on identified strengths, overcoming weaknesses, exploring opportunities and minimising threats (BOEM) were formulated. Action plans were designed to facilitate the implementation of the guidelines by midwives and managers, displayed in Tables 1 and 2.

The strengths and opportunities were enablers for the organisation to achieve its objectives, whereas weaknesses and threats were regarded as harmful for the organisation to achieve its goals, and these must be manipulated.^20,21,22^

From Table 1, the first strategy indicated as a priority was the availability of human resources with 74 scores, it included the availability of MHCM and OMN (as appointed for the position). Regardless of their availability as per appointment, they were not always available in the facility to supervise and monitor midwives in the provision of maternal healthcare services. An action that will facilitate midwives’ implementation of maternal healthcare guidelines was indicated. For example, the development of scheduled site visits by MHCM will assist in monitoring and evaluating maternal healthcare services and enhancing support. Programme integration for OMNs to have the opportunity to supervise midwives and come up with strategies to deal with problems experienced by midwives when rendering maternal healthcare services was another thought. Hiring midwives to complement the available staff was thought for midwifes to have possibly made use of maternal guidelines. At present, one midwife will render maternal healthcare services alone day and night, which is having a detrimental effect on maternal healthcare services. Another action to enhance the availability of human resources was the clubbing of PHC facilities. Certain PHC facilities are to be grouped with midwives selected from other facilities to come and render maternal healthcare services, especially at night in one facility. This was thought to reduce the shortage of midwives and promote 24-h maternal healthcare services, hence the implementation of maternal health care guidelines. Knowledge deficit was ranked as number two by 70 scores, as a result, advanced midwives or two midwives who will assist each other will be allocated during the management of pregnant women as they will have time to peruse the guidelines and refer to protocols when encountering obstetric emergencies. Capacitating midwives with knowledge through workshops, in-service training and other forms of training related to maternal health will aid in the implementation of maternal guidelines correctly. Third priority identified was provision of maternal healthcare services with 69 scores, where, for example, the following was indicated: Referral of pregnant women when a need arise, offering of HIV services to pregnant women and purchasing of ambulance designated for maternal healthcare services.

Table 2 depicts the ranking of strategies 4 to 7 with total scores from 68 to 63. These strategies have to do with the exploration of opportunities and minimising threats. Researchers indicated priority strategy number four as quality assurance, where OMNs conducted monthly facility assessments and peer reviews. Though client satisfaction surveys were not done, an action to develop standardised survey forms was indicated. As such, midwives will be able to identify the strengths and weaknesses in providing maternal healthcare services, in turn enhancing the implementation of maternal healthcare guidelines. The fifth strategy was the availability of PMTCT and maternity care guidelines utilised by midwives, hence facilitating maternal healthcare services. To promote the implementation of maternal healthcare guidelines, the community plays an important role. As such, there is a need to involve them in the care provided to their family members. Community involvement was ranked number 6, where family members, especially mothers-in-law and spouses, are encouraged and allowed to accompany pregnant women for antenatal care services and for delivery. In South Africa, reduction of waiting times is one of the items indicated in the national core standards; as such, it was rated number seven by the group. To minimise its threats, consolidation of maternal healthcare registers was thought of as it was discovered that there were a lot of registers that midwives complete for every pregnant woman, hence spending more time with one pregnant woman. The NGT team also indicated appointment strategies to reduce waiting times.

## Discussion

### Priority strategies to building on the strength and overcoming weaknesses

The strengths and weaknesses were discussed together. To build on strengths and overcome weaknesses, the following strategies were indicated and ranked according to their priority: Availability of human resources, knowledge deficit and provision of maternal health services.

### Human resources

Successful implementation of maternal healthcare services needs appropriate structures, including MHCMs, OMNs, midwives and other support staff. In this study, researchers identified that MHCMs and OMNs were available as strengths although there was a lack of support to midwives who provide maternal healthcare services. To build on it, researchers will work with MHCMs in developing scheduled site visits that will enhance the support visits to midwives working at PHC facilities. This will enable managers to monitor the implementation of maternal healthcare guidelines. According to Roets,^23^ their strategy suggested support visits to improve quality patient care. Maternal health care managers and OMNs work hand in hand to ensure that quality maternal healthcare services are delivered. Each PHC facility must be managed by an OMN. To build on the strength of OMN availability, an action of advocating for the integration of all programmes would be appropriate. This will allow OMNs to supervise and monitor midwives when rendering maternal healthcare services. Most of the time, OMNs were out of their facilities attending meetings. As a weakness, most OMNs were seconded to a higher post. An action of filling the position and giving them incentives will encourage them to supervise and monitor guidelines’ implementation by midwives. Supportive supervision as a strategy to enhance guidelines’ implementation was also indicated in a study conducted in Kenya, which was aimed at improving post-natal services.^23^ At the same time, Milford^24^ showed integration of services as a strategy to facilitate patients’ access to healthcare services.

### Knowledge deficit

The findings shown in Table 1 indicated the weakness of some midwives who had knowledge deficits and failed to adhere to maternal healthcare guidelines, which led to difficulties and frustrations when managing pregnant women with obstetric emergencies such as PPH and hypertensive disorders as pre-eclampsia or eclampsia. To overcome this weakness, advocating for the allocation of one advanced midwife to each PHC facility was thought of as this will assist with in-house in-service training of midwives. Arranging for continuous professional development, which includes ESMOE training to capacitate midwives with knowledge, was also supported by various authors.^23,24,25^ Training must cover all midwives to avoid the shift of responsibilities. Again, studying maternity guidelines when the facility is not busy was another action for improvement. Difficulty and frustrations can further be addressed by allocating two midwives to assist each other during the management of pregnant women. The provincial department of health will purchase manikins and equipment for midwives to enhance their skills by practising emergency obstetric simulation training (EOST) drills. This will further assist midwives in adhering to guidelines. This strategy is in line with the results of the implementation study conducted by Bhardwaj,^26^ where the impact of EOST drills was assessed. The study showed an improvement in midwives’ management of obstetric emergency conditions. However, continuous training is still needed, and it must be offered to all midwives.

### Provision of maternal healthcare services

This study included the PMTCT guidelines assessment to guide midwives during maternal healthcare services. The findings of this study revealed the availability of HIV counselling services in all PHC facilities. As such, the Saving’s Mother Report of 2014–2016 reported a reduction in some maternal death caused by opportunistic infections related to HIV and acquired immune deficiency syndrome (AIDS).^5^ Despite the availability of HIV counselling, the programme itself is associated with work overload. As suggested in this strategy by researchers, successful implementation of the PMTCT guidelines will require an increase in the number of human resources that deal with HIV and the availability of testing kits. Further action suggested for successful implementation required that registers completed during the care of pregnant women living with HIV need consolidation and a move to electronic records; this might reduce the burden of paperwork for midwives.^23^

Provision of maternal health services requires transferring pregnant women to the next level of care, which is the hospital, especially in obstetric emergencies such as PPH, pre-eclampsia or eclampsia. The barrier to facilitate this process was delayed ambulance response by ambulance services caused by the shortage of ambulances which was identified by researchers. One of the departmental core standards is for ambulance services to arrive at the PHC facilities within 60 min of being requested by midwives.^27^ To minimise this weakness, an act of writing motivational word to the Department of Health (DoH) to purchasing more ambulances designated for maternal healthcare use only was suggested and each local area to have at least one ambulance at their disposal. This will assist midwives when in need of transferring pregnant women to the hospital, hence facilitating the implementation of the guidelines.

Hypertensive disorders in pregnancy were rated number one as the most common cause of maternal death in African countries.^4,28^ As such the provision of *maternal healthcare services* and management of pregnant women with pre-eclampsia or eclampsia need the administration of magnesium sulphate to decrease the morbidity and mortality rate. The findings of the study indicated that in some of the PHC facilities, this drug was insufficient. Various researchers also identified this weakness of insufficient drugs although they were not related to magnesium sulphate including a maternal health policy strategy developed in Nigeria and middle-income countries.^25,29^ As an action in this current strategy, researchers thought of advocating these facilities to be issued with 40 vials as the standard is 20 vials per facilities, especially with facilities that are far away from the hospital. Again, a suggestion of daily monitoring and use of standardised drug register will alert midwives if they are running out of stock. Another suggestion made by the researcher is the inclusion of magnesium sulphate and uterotonics on the cell phone tracer reporting system to ensure its availability, this might aid in the reduction of MMR caused by hypertensive disorders in pregnancy as midwives will be able to administer the drugs over time, hence saving the lives of women and their newborns.

*Maternal healthcare services* must be available all the time; hence, an action of all PHC facilities to render 24-h services was thought of as some were operating during the day only. As such, researchers thought of writing motivational word for the district managers to increase security and provide necessary resources such as more midwives and offer psychological support for the service to be accessible and available all the time. Another thought that will ensure access and availability of the services was the issue of building maternity hospitals with maternity homes and establishing maternal obstetric units (MOUs) for pregnant women to utilise. This will also address the weakness of failure by pregnant women to honour their follow-up visit and the long distances that they had to travel to access the healthcare services. A systemic review of various strategies indicated maternity waiting home as a way to promote the utilisation of maternal health services.^29^ Researchers disseminate findings to the DoH and district executive managers to address long-distance travel to PHC facilities by purchasing park homes that might be used in communities living far away from PHC facilities. This might promote access to maternal healthcare services by pregnant women.

### Priority strategies and their actions in exploring opportunities and minimising threats

Table 2 indicates the results of strategies on opportunities and threats and their action plans. The researchers presented action plans to explore opportunities and minimise threats. The findings of the study indicated strategies of opportunities and threats in their rating order as follows: Quality assurance, availability of maternal guidelines, community involvement and reduction of waiting times.

### Ensuring quality assurance

Table 2 indicates the opportunity to ensure that quality maternal healthcare services are provided all the time; as such monthly facility, supervisory assessments and peer reviews are conducted mainly by OMNs of different PHC facilities. They check records, including maternal healthcare records, for proper care and identification of gaps. This improves the provision of maternal services. To further enhance the quality of maternal healthcare services, action on monthly perinatal meetings conducted by the two districts was emphasised as the meetings equip midwives with knowledge and skills. This strategy was also supported by other researchers who indicated that sessions would assist midwives in improving the healthcare services rendered to mothers.^23^ Almost all the facilities failed to conduct client satisfaction surveys and clinical audits. An action sought to address this threat is to work with the district executive managers to ensure the availability of a standardised client satisfaction survey in each PHC facility to improve the health of our nation. Another action indicated by researchers is to encourage midwives to ensure that each pregnant woman completes this survey on discharge to improve maternal healthcare services. This strategy was similar to the one developed by Roets^23^ to enhance the lives of mothers.

The survey data are analysed and are used to formulate corrective measures. Researchers corroborated that failure of conducting surveys and clinical audits could be a threat.^24,27^ The OMNs and the second-in-charge are facilitated to review the pre- and postnatal maternity records fortnightly. This will assist in identifying gaps and further drawing a strategy to improve the knowledge and skills of midwives. Furthermore, to enhance the quality of care rendered to pregnant women, researchers indicated that mom Connect programme is available; hence researchers thought of facilitating the incorporation of cell phone reminders in this programme. This will improve adherence to follow-up care by pregnant women, as such complications can be identified earlier and treated.

### Availability of prevention of mother-to-child transmission guidelines, maternity care guidelines and protocols

Researchers identify the availability of maternal guidelines and protocols that guide midwives in caring for pregnant women – advising midwives to develop a positive attitude towards the guidelines as a tool that shows their management. In collaboration with the National Department of Health, they must ensure that all PHC guidelines are packaged electronically and made available to all midwives to facilitate their implementation and improve quality care. Other researchers also indicated the use of technologies such as e-learning as a strategy with effective results.^24,26^

### Community involvement

Community involvement is crucial in maternal healthcare service delivery; without the community, the facility will not function properly as its inputs play an essential role in community mobilisation. Most of the PHC facilities allow accompaniment of pregnant women by family members, especially during delivery. An action to facilitate and arrange meetings for traditional leaders, clinic committee members and home-based carers to influence the community on accompanying pregnant women to the PHC facilities, especially for delivery was indicated. This form of support by family members will enable midwives to implement the maternal guidelines with ease because some pregnant women when they experience labour pains, refuse to listen to the advice given by midwives. According to Ezeonwu,^30^ family and spouse involvement in the pregnant women’s care and accompaniment for delivery also called public enlightenment was thought to improve maternal care outcomes. Another form of involving the community lies in the integration of community structures such as home-based carers, local churches, life orientation teachers for both primary and secondary levels on matters related to maternal healthcare services. Actions to train home-based carers on maternal health-related matters about pregnant women include measurement of blood pressure, urinalysis, observation of danger signs which needs urgent referral and superficial knowledge of maternal healthcare guidelines. Researchers thought of organising training in a form of workshops for life orientation teachers so that they have a superficial understanding of the maternal healthcare guidelines to handle pregnant students at schools as the department allows a pregnant girl child to continue with their studies.^31^

### Reduction of waiting times

One of the ministerial priorities is reducing the time where a patient is supposed to wait where the healthcare service is offered.^27^ Each PHC facility has the minimum and maximum waiting period pasted inside the facility for patients to know. Waiting times reduction will be addressed by the consolidation of registers, especially amongst pregnant woman visiting for the first time on whom several procedures will be carried out, including HIV counselling and testing. Another action is the use of an appointment register which will promote utilisation of maternal healthcare services as patients won’t wait for a more extended period. The establishment of park homes in communities that do not have an access to a PHC facility within 5 km radius will reduce the overflow of patients to PHC facilities. In the long run, it will facilitate the use of guidelines by midwives as the number of patients will be reduced to normal and improve maternal healthcare services.^32^

### Limitations of the study

The developed strategies focus on the selected maternal health conditions and may not be applied to other medical conditions and other settings. The NGT process was completed in one day and some of the strategies might have been elaborated on further if more time was available.

## Conclusion

This study aimed on developing and prioritising strategies to improve midwives implementation of maternal healthcare guidelines. The NGT group was invited to be part of the current strategies development. The strategies identified were rated according to their priorities following SWOT analysis. The researchers identified SWOT that influenced the provision of maternal healthcare services. The use of the SWOT analysis matrix and BOEM helped the NGT group as researchers to reach their goal of developing and prioritising the current strategies. Strategies such as the availability of human resources, knowledge deficit and provision of maternal health services were identified as both strengths and weaknesses. In contrast, quality assurance, lengthy waiting times, availability of maternal guidelines and community involvement were opportunities and threats. Researchers were able to formulate actions that could be used to build on identified strengths, overcome weaknesses, explore opportunities and mitigate the threats. Although experts in maternal healthcare services validated this strategy, it is yet to be implemented. Its implementation might lead to further refinement of the current strategies as such provision of maternal healthcare will improve which is anticipated to reduce the MMR. Researchers recommended that all the stakeholders involved in maternal healthcare services starting from MHCMs at the national, provincial, district office and District Executive Manager (DEM), OMNs and midwives at PHC facilities familiarise themselves and implement the developed strategies. To build on the strengths and overcome weaknesses, the DoH at national, provincial and district levels must provide resources for midwives. In turn, midwives must utilise the resources during the provision of maternal healthcare services, this will improve the implementation of maternal guidelines. Further research on the effectiveness of the implementation of the current strategies is recommended. The strategies could reduce MMR, hence achieving the third goal of Sustainable Development Goals by 2030.
